# Participant Evaluation of Blockchain-Enhanced Women’s Health Research Apps: Mixed Methods Experimental Study

**DOI:** 10.2196/65747

**Published:** 2025-03-25

**Authors:** Madelena Y Ng, Jodi Halpern, Olivia Shane, Tina Teng, Michael Nguyễn, Casey Ryan Alt, Anaïs Barthe Leite, Sean Moss-Pultz, Courtney R Lyles, Coye Cheshire

**Affiliations:** 1 School of Public Health University of California, Berkeley Berkeley, CA United States; 2 Department of Medicine (Biomedical Informatics) Stanford University Stanford, CA United States; 3 Haas School of Business University of California, Berkeley Berkeley, CA United States; 4 Bitmark, Inc Taipei Taiwan; 5 Department of Public Health Sciences University of California, Davis Davis, CA United States; 6 Department of Medicine University of California, San Francisco San Francisco, CA United States; 7 School of Information University of California, Berkeley Berkeley, CA United States

**Keywords:** blockchain technology, privacy, trust, data control, data ownership, digital health study, user-centered design, user experience, mHealth, mobile health, women’s health, reproductive health, data sharing, research participation, bioethics

## Abstract

**Background:**

Blockchain technology has capabilities that can transform how sensitive personal health data are safeguarded, shared, and accessed in digital health research. Women’s health data are considered especially sensitive, given the privacy and safety risks associated with their unauthorized disclosure. These risks may affect research participation. Using a privacy-by-design approach, we developed 2 app-based women’s health research study prototypes for user evaluation and assessed how blockchain may impact participation.

**Objective:**

This study aims to seek the perspectives of women to understand whether applications of blockchain technology in app-based digital research would affect their decision to participate and contribute sensitive personal health data.

**Methods:**

A convergent, mixed methods, experimental design was used to evaluate participant perceptions and attitudes toward using 2 app-based women’s health research study prototypes with blockchain features. Prototype A was based on the status quo ResearchKit framework and had extensive electronic informed consent, while prototype B minimized study onboarding requirements and had no informed consent; the mechanisms of how the contributed data flowed and were made pseudonymous were the same. User evaluations were carried out in February and March 2021 and consisted of a think-aloud protocol, a perception survey, and a semistructured interview. Findings were mapped to the technology acceptance model to guide interpretation.

**Results:**

We recruited 16 representative female participants from 175 respondents. User evaluations revealed that while participants considered prototype B easier to use on intuitive navigation (theme 1) of specified tasks and comprehension (theme 2) of research procedures, prototype A trended toward being perceived more favorably than prototype B across most perception survey constructs, with an overall lower level of privacy concern (mean [SD]: 2.22 [1.10] vs 2.95 [1.29]) and perceived privacy risk (2.92 [1.46] vs 3.64 [1.73]) and higher level of perceived privacy (5.21 [1.26] vs 4.79 [1.47]), trust (5.46 [1.19] vs 4.76 [1.27]), and usability (67.81 [21.77] vs 64.84 [23.69]). Prototype B was perceived more favorably than prototype A with perceived control (4.92 [1.32] vs 4.89 [1.29]) and perceived ownership (5.18 [0.59] vs 5.01 [0.96]). These constructs, except for perceived ownership, were significantly correlated with behavioral intention to use the app (*P*<.05). Participants perceived the usefulness of these prototypes in relation to the value of research study to women’s health field (theme 3), the value of research study to self (theme 4), and the value of blockchain features for participation (theme 5).

**Conclusions:**

This study provides nuanced insights into how blockchain applications in app-based research remain secondary in value to participants’ expectations of health research, and hence their intention to participate and contribute data. However, with impending data privacy and security concerns, it remains prudent to understand how to best integrate blockchain technology in digital health research infrastructure.

## Introduction

Blockchain, or distributed ledger technology, has capabilities that can alleviate persistent concerns in digital health research [1-3]. A blockchain is a decentralized database that maintains a growing list of records or transactions using cryptographically linked blocks [1,2]. The blockchain requires consensus across all peers or “nodes” of the network before a block can be added, thus enabling the validation of transactions in a decentralized manner [1,2]. Once recorded on a blockchain, transactions are immutable (cannot be changed or deleted), existing in a verifiable and auditable manner that fosters transparency and trust [1,3]. While blockchain is best known as the underlying technology of cryptocurrencies like Bitcoin, the application of its properties in the health research sector can transform the ways we share, access, and use data [1,4].

For digital health research, vast quantities of health data captured through wearables, mobile health (mHealth) apps, smartphones, and Internet of Things devices coupled with clinical and “omics” data hold valuable insights into individuals’ health and behavior. As these large datasets or “big data” grow in scope, they also become more susceptible and vulnerable to privacy risks (eg, data breaches, privacy violations, and unauthorized access) that can cause undue harm to participants, organizations, and society [5]. Health research is also increasingly conducted outside the purview of academic health care institutions, which must comply with stringent regulations (eg, Health Insurance Portability and Accountability Act and human research subject protections) to safeguard patient privacy and ensure ethical research by independent entities unencumbered by these requirements. For example, many marketplace mHealth apps that use user data for health research or algorithm training purposes lack formal informed consent processes or are unclear about how to opt in or opt out of research, further challenging the ethical norms of human subject research [6].

These risks are salient in women’s health research, a field that is understudied with large existing health disparity gaps [7] and historic injustices that left lingering mistrust in biomedical interventions [8]. A boom of “femtech” (female- or women-centered technologies) tools are enabling women to regularly report and track data related to menstruation, pregnancy, fertility, pelvic or sexual health, and cancers, among other relevant topics [9-11]. While these data offer immense opportunities to advance women’s health, the data are also inherently sensitive, intimate, and taboo in certain sociocultural contexts [9]. Exposure can cause women potential harm such as shame, discrimination, and even violence [9]. Women’s willingness to share personal health data may be further dampened by the 2022 US landmark *Dobbs v Jackson Women’s Health Organization* case that overturned the privacy precedents established by *Roe v Wade* [12,13]*.* Privacy concerns and discomfort with data sharing were also reported as reasons for the mHealth app abandonment [14]. Thus, the presence of robust data security safeguards is critical to the outlook of participation, trust, and quality of data generated for health research.

Blockchain can reinforce the security of digital health study infrastructure and offer participants greater protection over their data. Data stored on the blockchain can be analyzed but remain private, as the owner is pseudonymous, and a private key is needed to access their data [1,3]. Participants can grant researchers access to select personal health data by initiating transactions signed by their private key, thereby exercising control and ownership over their data in a digital environment [1-4]. This process would require participants to be active contributors of their data, with each transaction on the blockchain serving as a record of their consent. Marketplace health tools can use this approach to be resilient toward evolving data-sharing regulations; however, it remains to be seen how users perceive its value and impact in driving women’s health research.

This study sought the perspectives of women, centering on their perceptions and attitudes, to understand how applications of blockchain technology in app-based digital research affect their decision to participate and contribute data. In collaboration with Bitmark Inc, a domain-agnostic blockchain applications company, we took a “privacy-by-design” [15] approach in creating 2 app-based women’s health research study prototypes—prototypes A and B. Prior to the launch of a “live” or in-production app, rigorous user testing on prototypes helps uncover core real-world matters that may affect adoption. We, therefore, carried out a convergent, mixed methods, experimental study of the 2 prototype variants with 16 female participants between February and March 2021. This study aims to show the complexity of human and sociotechnical considerations that arise with blockchain integration into digital health research infrastructure.

## Methods

### Study Design

**Figure 1:**
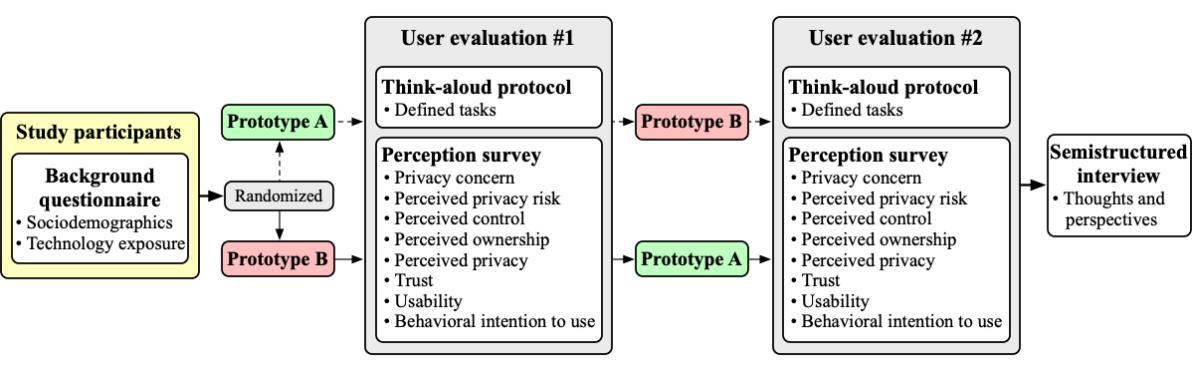
Summary of the convergent, mixed methods, experimental design.

A convergent, mixed methods, experimental design [16] was used to ascertain the data for our study (Figure 1). The experimental portion consisted of a crossover trial design where female participants were randomly assigned to either test (1) prototype A first, followed by prototype B, or (2) prototype B first, followed by prototype A. This served to address any potential spillover effects from testing one prototype first over the other. The mixed methods portion consisted of (1) usability tests for each prototype with a think-aloud protocol (during testing) and perceptions survey (after testing) and (2) a semistructured interview to gauge a more nuanced understanding of participant thoughts and perspectives.

### Industry Partnership

A partnership with Bitmark Inc produced 2 unique prototypes guided by real-world blockchain applications for our comprehensive evaluation. The Bitmark system is built on a public blockchain, which is a digitized, decentralized, and public ledger that records all occurring data transfers. Bitmark issues digital property titles, known as “bitmarks,” for a person’s digital data and records them on the blockchain. In the context of digital health research, this system enables participants to contribute their “bitmarked” data to researchers pseudonymously. Bitmark Inc provided technical and design support for the prototypes.

### Prototype Development and Description

Development cycles were carried out between study researchers and Bitmark engineers to design the 2 baseline prototypes: prototype A and prototype B. These prototypes each present a hypothetical digital research infrastructure where blockchain capabilities were differentially operationalized and conveyed to participants. Prototype A proposed a more familiar process of carrying out app-based digital research where an extensive electronic informed consent (eIC) process exists, and participant health data were actively transferred from the participant to the researcher; the ResearchKit framework from Apple served as the basis for the design. Prototype B envisioned a more radical process of app-based research participation where informed consent was not required, and participant health data were stored in a “data vault” for open aggregate analyses by the research community. The features of both prototypes, including their similarities and differences, are listed in Multimedia Appendix 1. The prototypes were produced as a series of high-fidelity wireframes and presented using Figma (Figma Inc), a design and prototyping software tool. Relevant wireframe designs and brief descriptions are provided in Multimedia Appendix 2.

### Participant Recruitment and Sample

Women, aged 18 years or older, were recruited through the University of California, Berkeley (UC Berkeley) Experimental Social Science Laboratory (Xlab) for user evaluations. A recruitment email with a background questionnaire, administered through the survey platform Qualtrics (Qualtrics International Inc), was sent to Xlab’s participant pool to obtain sociodemographic, smartphone, and health app use information from interested respondents (Multimedia Appendix 3). Email recruitment yielded a total of 175 respondents who completed the questionnaire.

We assessed the eligibility of the 175 respondents and purposively sampled across 4 groups to obtain a representative sample of 16 participants for user evaluations (Multimedia Appendix 4). Our 4 groups consisted of (1) White, non-Hispanic women of higher socioeconomic status (SES), (2) White, non-Hispanic women of lower SES, (3) women of color with higher SES, and (4) women of color with lower SES. Women of color were defined as those respondents who did not identify as White and non-Hispanic (eg, non-Hispanic Black, non-Hispanic Asian, Hispanic, or Latino). SES was defined by either above (higher SES) or below (lower SES) 100% area median income (AMI; adjusted to San Francisco Bay Area standards) or presence (higher SES) or lack of (lower SES) college-level education. We took an intersectional approach in determining the 4 groups, which accounted for differences in the association between certain sociodemographic variables (eg, race or ethnicity, income, education) and the likelihood of participating in health research [17]. Within each of these groups, we also considered the respondents’ level of digital health research and technology exposure. A higher level of digital health literacy was defined as those who had either previously joined a digital research study or ever used a women’s health app.

### Study Setting

All user evaluation sessions were conducted in English and remotely through Zoom (Zoom Video Communications) from February to March 2021. Sessions were video and audio recorded with participant consent. Following our study design (Figure 1), participants were randomized to a testing arm to evaluate the prototypes. We first asked participants to complete 3 tasks in 1 prototype while thinking aloud (think-aloud protocol), followed by completing a perception survey about the prototype; these procedures were repeated for the next prototype. Participants accessed each prototype through a Figma link and shared their screen in Zoom during the session recording. Participants completed a perception survey for prototypes A and B after each think-aloud protocol. The total mean (SD) time to complete both think-aloud protocols was 39 (10) minutes; recordings were paused during the completion of the perception surveys. After completing user testing for both prototypes, we conducted a semistructured interview with participants to capture their experiences and thoughts. The total mean (SD) time of the interview was 13 (4) minutes. Participants took approximately 60-90 minutes to complete the entire evaluation session. We employed Rev (Rev.com Inc), a reputable commercial human transcription service, to transcribe session audio recordings.

### Data Collection Instruments

We describe the data collection instruments used in our mixed methods, experimental design (Figure 1) in detail below. All data collection instruments can be found in Multimedia Appendix 3.

#### Background Questionnaire

A background questionnaire gathered sociodemographic and general technology exposure information (ie, usage of smartphone, app-based health research study, or women’s health-related app) from interested study respondents. Respondents accessed the questionnaire through a Qualtrics link in the recruitment email.

#### Think-Aloud Protocol

The usability of the prototypes was assessed using a think-aloud protocol with a defined interaction approach. Defined tasks are specified for the participant to complete. Participants were asked to “think-aloud” or verbalize their thought processes while they completed (or tried to complete) each task. Participants were asked to carry out the following three tasks: (1) create an account or register in the app, (2) join a women’s health research study, and (3) contribute health data to the study. These tasks were selected because they form the basis of app-based digital health research participation, and their usability can influence early attitudes toward intent to participate.

#### Perceptions Survey

Our perception survey measured eight constructs: (1) privacy concern, (2) perceived privacy risk, (3) perceived control, (4) perceived ownership, (5) perceived privacy, (6) trust, (7) usability, and (8) behavioral intention to use the app. Measurement items for each construct were gathered and adapted from validated surveys or scales in the literature [18-23]. Items were selected to capture participants’ perceptions in relation to their intention to use either prototype. Items are assessed on a 5- or 7-point Likert scale ranging from 1=strongly disagree to 5 or 7=strongly agree, depending on the construct. Summative scales or a calculated score (for usability only) were produced to represent each construct. Participants accessed the perceptions survey through a Qualtrics link after completing the think-aloud protocol for each prototype.

#### Semistructured Interview

A semistructured interview helped gather more in-depth thoughts or perspectives from participants that may not have been captured through the task-based activities or surveys from the testing session. The interview guide was informed by salient questions surrounding our study topic and was purely exploratory.

### Data Analysis

We describe the analysis approach for the data of each instrument.

#### Background Questionnaire

Descriptive statistics were used to summarize the sociodemographic and general technology exposure characteristics of the women who completed the entire user evaluation of prototypes A and B.

#### Think-Aloud Protocol

The study lead (MYN) reviewed the cognitive interviews, audiovisual recordings during user testing, and corresponding transcript data to ascertain information about task performance. For each task, it was determined whether the participant (1) completed the task with ease, (2) completed the task with help, or (3) did not complete the task. Task completion was defined as the correct completion of the task and participant acknowledgment. Ease of completion was defined by whether the participant experienced a low versus high level of difficulty or confusion in completing the task; this was determined based on a combination of time elapsed, clicks (trial-and-error), and expressed emotions (familiarity, excitement, frustration, and doubt) until completion acknowledgment. Those who completed the task with ease were generally more confident in stating their completion, while those who completed the task with help were less certain or had lingering concerns. Task incompletion was defined by incorrect completion of the task (eg, incorrect stop points) or an explicit statement that (1) they did not believe they completed the task or (2) they would not have proceeded to complete the task under usual circumstances.

#### Perceptions Survey

We carried out reliability analyses for each construct’s set of measurement items to determine their “internal consistency” or how well they go together [15]. Reliability was determined using Cronbach α; a value of more than 0.70 means high reliability, 0.35 to 0.70 means acceptable reliability, and less than 0.35 means low reliability. Summative scales were created for each construct, with the exception of usability. For the usability construct, we generated a System Usability Scale (SUS) score between 0-100 following the scale’s validated process; an SUS score of 68 is considered average. Pearson correlation analyses were performed to determine the magnitude and direction of the linear relationship between all the perception survey constructs and behavioral intention to use the app (outcome variable) for prototypes A and B; *P* values of <.05 were considered statistically significant. An absolute Pearson correlation coefficient of more than 0.70 means strong to very strong correlation and 0.40 to 0.69 means moderate to good correlation [24]. Due to the small sample size, we used the Wilcoxon rank-sum (Mann-Whitney *U*) test, a nonparametric test of mean difference*,* to determine whether there were significant differences between each construct’s mean or score in prototype A versus prototype B. The significance level for all tests was set at the .05 level (5%). All statistical analyses were conducted using STATA (version 16.0; StataCorp).

#### Semistructured Interview

Three coders (MYN, OS, and TT) used a thematic analysis approach to process transcripts in MaxQDA (VERBI Software), a qualitative data analysis software program. First, the study lead (MYN) generated initial codes from the raw semistructured interview data using a mixed deductive-inductive approach. Deductive codes were generated based on broad content areas (eg, perceptions about blockchain, research participation, and health data contribution) that corresponded to the interview questions. Inductive codes emerged from close reading of the interview transcript data. Second, the 3 coders (MYN, OS, and TT) used an initial codebook to independently code a subset of transcripts. The coding team discussed discrepancies until a consensus was reached. The initial codebook was iteratively refined throughout this process. The study lead (MYN) reapplied the updated coding frame to all coded transcripts to attain alignment. Finally, identified emergent concepts and connections were examined to identify themes relevant to the use of an app-based women’s health research study. Key themes were mapped to the constructs of the technology acceptance model (TAM; ie, perceived ease of use and perceived usefulness) to explore how they relate to participants’ attitudes and behavioral intention to use an app-based research study with integrated blockchain features [25,26].

### Ethical Considerations

Human subject ethical review approval for this study and all procedures were obtained from the UC Berkeley Institutional Review Board (2021-02-14021). All participants provided verbal consent upon reviewing the study information and before taking part in study procedures. Participant data are safeguarded and stored on encrypted devices with minimal identifiers. Respondents to the background questionnaire were entered into a US $15 raffle. Those who participated in the full evaluation session were paid US $30 to US $35 for their time. We followed guidelines from the Checklist for Reporting Results of Internet E-Surveys (CHERRIES) for the perception survey and the Consolidated Criteria for Reporting Qualitative Studies (COREQ) [27,28] for the semistructured interview.

## Results

### Participant Characteristics

A total of 175 respondents completed the background questionnaire in the recruitment email. Using purposive sampling, we reached out to respondents until we attained a balanced representation of 4 participants each for the aforementioned 4 groups (Multimedia Appendix 4). These 16 women participated in the user evaluation of prototypes A and B, which consisted of a think-aloud protocol (task performance and cognitive interview) and perception survey for each prototype, followed by a concluding semistructured interview.

Participant characteristics are displayed in Table 1. For race or ethnicity, of the 16 women, 8 (50%) identified as non-Hispanic White and 8 (50%) identified with other racial and ethnic groups. Participants’ average age was 29 years, with 8 (50%) women in the 18-29 years age group, 6 (38%) in the 30-39 years age group, and 2 (13%) in the 40-49 years age group. We did not have any respondents in the 50-59 years or 60 years or older age groups. For education level, 6 (38%) women did not have a college-level degree while 10 (63%) had a college-level degree or above. For AMI, 9 (56%) women were below 100% AMI, while 7 (44%) were above 100% AMI. In terms of having previous exposure to the study topic, 8 (50%) women have either ever used a women’s health app or ever joined a digital research study, while 8 (50%) have done neither. Most participants (15/16, 94%) have owned a smartphone for over 5 years and indicated they mostly (14/16, 88%) use their smartphones for less than 5 hours each day.

**Table 1:** Characteristics of women participants who evaluated prototype A and prototype B (N=16).

Characteristic			Participants, n (%)
**Race or ethnicity**			
	Asian, non-Hispanic	5 (31)	
	Black, non-Hispanic	1 (6)	
	Hispanic or Latino	2 (13)	
	White, non-Hispanic	8 (50)	
**Age group (years)**			
	18-29	8 (50)	
	30-39	6 (38)	
	40-49	2 (13)	
**Education**			
	Some college, no degree	4 (25)	
	Associate’s degree	2 (13)	
	Bachelor’s degree	6 (38)	
	Master’s degree	2 (13)	
	Doctoral or professional degree	2 (13)	
**AMI^a^** (%)			
	Less than 50	4 (25)	
	50-99	5 (31)	
	100-149	2 (13)	
	150-200	1 (6)	
	More than 200	4 (25)	
**Familiarity with study topic**			
	Ever use a women’s health app	7 (44)	
	Ever join a digital research study	1 (6)	
	Neither	8 (50)	
**Smartphone ownership (years)**			
	3-5	1 (6)	
	More than 5	15 (94)	
**Smartphone usage per day (hours)**			
	1-2	6 (38)	
	3-4	8 (50)	
	More than 5	2 (13)	

^a^AMI: area median income.

### Think-Aloud Protocol

The 16 participants’ task performance of the 3 tasks for each prototype is summarized in Table 2. We identified a key theme from the participants’ cognitive interviews relevant to the perceived ease of use construct.

**Table 2:** Task performance identified through usability testing (N=16).

Task performance		Prototype A, n (%)				Prototype B, n (%)		
		Task 1^a^	Task 2^b^	Task 3^c^	Task 1^a^		Task 2^b^	Task 3^c^
**Completed**								
	With ease	5 (31)	0 (0)	16 (100)	4 (25)		11 (69)	15 (94)
	With help	4 (25)	16 (100)	0 (100)	10 (63)		5 (31)	0 (0)
**Not completed**		7 (44)	0 (0)	0 (0)	2 (13)		0 (0)	1 (6)

^a^Task 1: Create an account or register in the app.

^b^Task 2: Join a women’s health research study.

^c^Task 3: Contribute health data to the study.

### Theme 1: Intuitive Navigation

Overall, the task performance and cognitive interviews provided important context about intuitive navigation in facilitating participants’ perceived ease of use with the health research study app. Intuitive navigation was assessed in our study based on whether participants were able to (1) definitively determine whether an account was created (task 1), (2) locate the research study within the app in a straightforward manner (task 2), and (3) easily contribute health data to the research study (task 3).

Task 1 was easier to complete in prototype B than in prototype A. Although more participants completed task 1 overall in prototype B (14/16, 88%) than in prototype A (9/16, 56%), many acknowledged completion with a level of help. The findings from task 1 suggest that the concept of a pseudonymous account is still quite foreign to users and the prototypes can better expound on its significance.

Task 2 was easier to complete in prototype B than in prototype A. Although all participants completed task 2 in both prototypes, more completed the task with help in prototype A (16/16, 100%) than in prototype B (5/16, 31%). The findings from task 2 stress the importance of a clear and intuitive interface to effectively direct participants from account creation to the research study.

Task 3 was largely completed with ease by participants in both prototypes, with 16 (100%) in prototype A and 15 (94%) in prototype B. The findings from task 3 show that participants are familiar with the concept of data contribution to research in a remote setting.

### Perceptions Survey

The quantitative assessments of the 16 participants’ perceptions toward each prototype are presented in Table 3. Reliability analyses revealed that each construct’s set of measurement items had Cronbach α values over 0.70, which indicates high reliability.

**Table 3:** Mean values and intercorrelations between perception survey constructs and behavioral intention to use the app (N=16).

Perception constructs	Prototype A				Prototype B		
	Cronbach α	Mean (SD)	*r*	Cronbach α		Mean (SD)	*r*
Behavioral intention to use app^a^	0.79	3.52 (0.68)	1.00	0.89		3.33 (1.04)	1.00
Privacy concern^a^	0.96	2.22 (1.10)	–0.61^d^	0.96		2.95 (1.29)	–0.59^d^
Perceived privacy risk^b^	0.90	2.92 (1.46)	–0.61^d^	0.96		3.64 (1.73)	–0.52^d^
Perceived control^b^	0.85	4.89 (1.29)	0.73^d^	0.88		4.92 (1.32)	0.61^d^
Perceived ownership^b^	0.88	5.01 (0.96)	0.26	0.77		5.18 (0.59)	0.17
Perceived privacy^b^	0.97	5.21 (1.26)	0.65^d^	0.96		4.79 (1.47)	0.66^d^
Trust^b^	0.95	5.46 (1.19)	0.62^d^	0.96		4.76 (1.27)	0.68^d^
Usability^c^	0.90	67.81 (21.77)	0.45	0.95		64.84 (23.69)	0.87^d^

^a^Assessed on a 5-point Likert scale ranging from 1=“strongly disagree” to 5=“strongly agree.”

^b^Assessed on a 7-point Likert scale ranging from 1=“strongly disagree” to 7=“strongly agree.”

^c^Calculated System Usability Scale score between 0-100.

^d^Denotes *P*<.05 and statistical significance in correlation with behavioral intention to use the app.

Pearson correlation analyses of the resultant summative scales and scores revealed insights into the 8 constructs, with a focus on the others’ relationship to behavioral intention to use the app (outcome variable). For both prototypes A and B, participants’ level of privacy concern (prototype A: *r*=–0.61; *P*=.01 and prototype B: *r*=–0.59; *P*=.02) and perceived privacy risk (prototype A: *r*=–0.61; *P*=.01 and prototype B: *r*=–0.52; *P*=.04) were shown to have a good negative correlation with the behavioral intention to use the app. In contrast, participants’ level of perceived control (prototype A: *r*=0.73; *P*=.001 and prototype B: *r*=0.61; *P*=.01), perceived privacy (prototype A: *r*=0.65; *P*=.01 and prototype B: *r*=0.66; *P*=.01), and trust (prototype A: *r*=0.62; *P*=.01 and prototype B: *r*=0.68; *P*=.004) with both prototypes were shown to have a good positive correlation with the behavioral intention to use the app. Usability was shown to only have a strong positive correlation with the behavioral intention to use the app in prototype B (*r*=0.87; *P*<.001). Perceived ownership of one’s health data in either prototype was not shown to have a significant correlation with the behavioral intention to use the app.

Overall, across all the perception constructs, a comparison of their mean scales or scores between prototype A and prototype B revealed no significant differences. However, prototype A trended toward being perceived more favorably than prototype B across most perception survey constructs, with an overall lower level of mean privacy concern (2.22, SD 1.10 vs 2.95, SD 1.29) and mean perceived privacy risk (2.92, SD 1.46 vs 3.64, SD 1.73) and a higher level of mean perceived privacy (5.21, SD 1.26 vs 4.79, SD 1.47), mean trust (5.46, SD 1.19 vs 4.76, SD 1.27), and mean usability (67.81, SD 21.77 vs 64.84, SD 23.69). Prototype B was perceived more favorably than prototype A with mean perceived control (4.92, SD 1.32 vs 4.89, SD 1.29) and mean perceived ownership (5.18, SD 0.59 vs 5.01, SD 0.96).

### Semistructured Interview

From 16 participants’ concluding interviews, we identified 4 more key themes relevant to perceived ease of use and perceived usefulness that drive participants’ overall attitude toward consistent use of the apps. All themes informed prototype improvement recommendations in Multimedia Appendix 5.

#### Theme 2: Comprehension

Participant comprehension of the research study and other technical safeguards (eg, blockchain applications) within the app facilitated their perceived ease of use. The presence of a formal eIC process in prototype A was helpful in informing and reassuring participants about the research study, hence fostering trust in health data contribution.

Prototype A just seemed more trustworthy design-wise and also because it really emphasized consenting… it gave more information on how the data was used.

User 3

However, the absence of an eIC process or other extensive explanatory content about blockchain technology (in prototype B) did not completely hinder one’s decision to participate and share health data.

As far as what information was being shared, and how secure I feel about it, I thought both apps did a good job… Prototype B was weaker on explaining security protocols, but it didn’t leave me feeling uncertain about the app or unwilling to share [data].

User 1

I’d probably contribute the same. Just because I trust the overall aim of what the study is trying to go for. I didn’t feel there was a difference in security. I just think the description of one was more convoluted than the other.”

User 5

#### Theme 3: Value of Research Study to Women’s Health Field

The perceived usefulness of the research study app is discussed in the following 3 themes. Participants were generally comfortable contributing to a majority of the requested women’s health data categories, including those typically considered to be more stigmatized. Most apprehensive thoughts centered around categories that required added acquisition costs (eg, ovulation test results, basal body temperature), additional clarification (eg, cervical mucus quality), or sensitivity tolerance (eg, sexual activity) to contribute data.

I'm just not a person that is too uncomfortable with a lot of these things because I just consider them all to be pretty human experiences that… a lot of women experience.

User 13

Overall, participants’ perceived value of their data contributions to the advancement of women’s health in research and medicine outweighed any discomforts or inconveniences with continued participation.

I feel like this information, given to the right institution or to the right study, has the potential to be instrumental and helpful for further generations of women for medical knowledge… I would love to see our medical knowledge expand in this area to be able to help more women, and for medicine… to be more equal.

User 7

#### Theme 4: Value of Research Study to Self

Participants expressed their continued participation depended on the extent of direct or indirect benefits to themselves, including positive feelings associated with seeing how their contributed data advanced study objectives and goals, their participation was appreciated and valued, and the presence of financial compensation. Regular updates about study progress and research findings were considered helpful for participants to assess whether continued participation was worth their time.

It would be nice to see how [my] information is being used... Not just knowing that I’m giving my information away with no real work to show for it. It’s not… set it to forget it, but you set it, and you’re not seeing results. It’s like, where’s my information going? What are they really using it for? So to see some updates of how that information is being used makes me want to keep using it. Makes me feel like... I’m contributing to these new findings... So [I would like to] see that update of: “Hey, with the recent health data being donated, we’ve realized that women from the ages 21 to 25 have an uptick in this, or there’s this pattern of this…”

User 14

A more granular understanding of how the research study is relevant to them and the return of insights about their contributed data is also a driver for their continued participation.

There’s also always: ‘What am I getting out of it?’ I’ve been more interested in signing up for studies that do compile your information and give it to you at the end, or tell you something about what they’ve learned about using your data.

User 8

Both prototypes did not intend to financially compensate participants for their time or data, aligning with the design of other app-based research studies. However, financial incentives were considered compelling for continued participation.

If it’s 10 minutes every day for 3 months, plus sharing this data and being thoughtful about my feedback, I’d want to know what the financial compensation is, if any… To participate in the study, I’d want it to be worth my time.

User 2

#### Theme 5: Value of Blockchain Features for Participation

Participants generally believed blockchain to be trustworthy and can enhance the privacy and security of contributed health data, but given their limited understanding of the technology, they could not definitively say whether its application in the study would affect their decision to participate.

On one hand, I don’t know if I’d want to really do a long-term study in general, but I guess knowing that the specific type of encryption technology, from my vague knowledge, I’ve heard is a relatively new and relatively well-respected technology, might make me more interested in contributing more data.

User 6

My doctor’s office… [has] an online portal where they keep all of my data and super intimate health information… I’m sure there’s special encryption that they use, but I don’t know the specifics of it. I just trust them. So I don’t know if necessarily having this blockchain technology is making me more likely [to participate]... but that’s probably because I don’t have a background of knowing anything about blockchain technology.

User 8

Also, given the novelty of blockchain technology in health care and its limited application, one participant expressed that it would take time for trust to be established and drive decision-making.

Typically, a new technology wouldn’t allure me until it had become more established, and maybe more generally accepted and widely used. I would be wary of it, just being the first time I’ve ever heard of it or used it. I don’t know that I would trust it right away.

User 1

### Integrated Conceptual Framework

**Figure 2:**
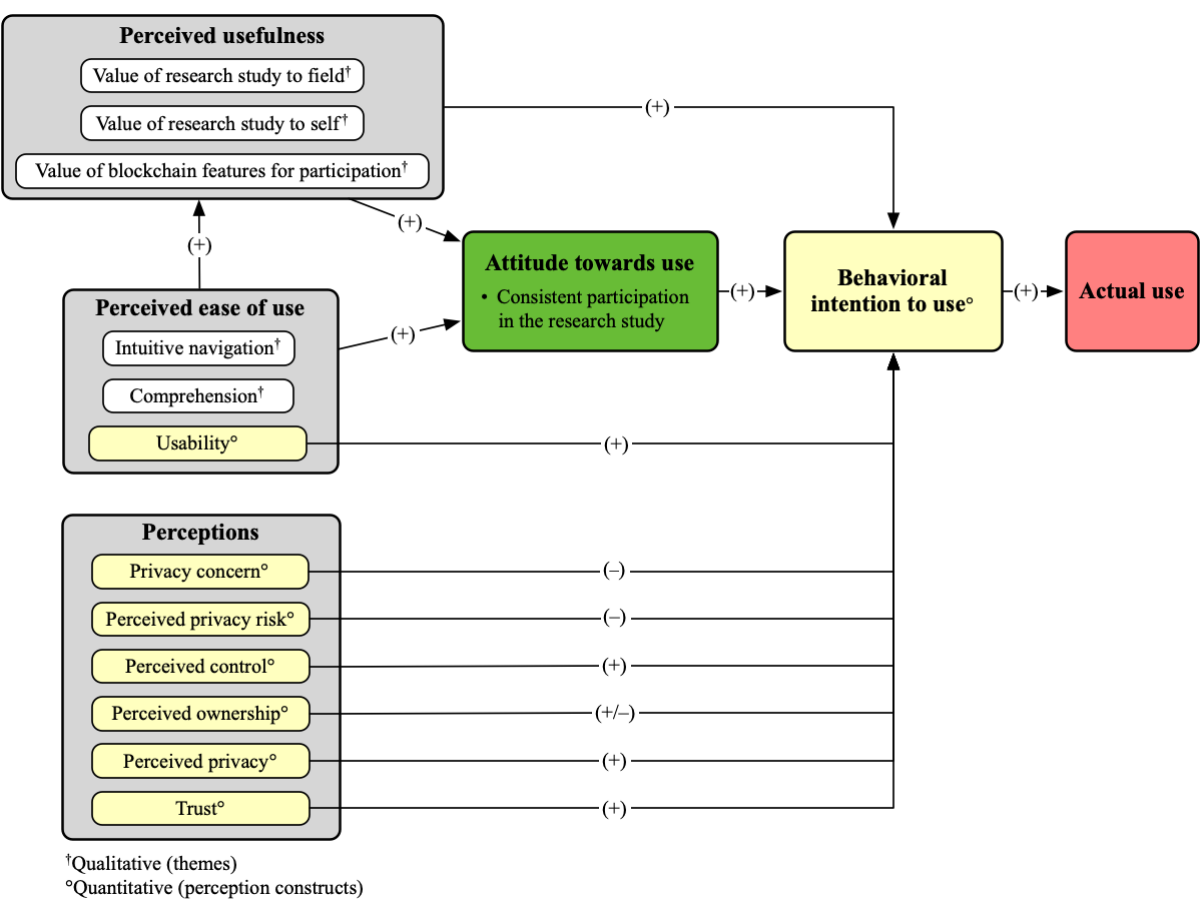
Integrated conceptual framework with qualitative and quantitative findings mapped to technology acceptance model constructs.

We mapped qualitative and quantitative findings from the user evaluations to TAM constructs. Figure 2 provides an overview of the integrated conceptual framework.

## Discussion

### Principal Findings

This study set out to understand potential women participants’ perceptions and attitudes toward the use of blockchain-enhanced women’s health research study apps. The prototypes proved to be effective representations for participants to ground their assessments. User evaluations reveal the limited influence of blockchain technology in contrast to a thoughtful research operations pipeline while fostering trust in digital health research participation. Our integrated conceptual framework (Figure 2) shows the nuanced factors that affect participation in this context.

Our study revealed 4 key findings on how digital health research teams and industry partners may approach the integration and use of emerging technology. First, while optimizing user interface design can directly improve the intuitive navigation of a prototype, it does not sufficiently address participant comprehension, which ultimately affects perceived usefulness. Recommendations in Multimedia Appendix 5 involve reworking and reorganizing the app content to better communicate key concepts about the study’s purpose and significance. These improvements reiterate the importance of a joint focus on optimizing technical design layouts alongside content usability, which aims to improve users’ uptake and processing of key information [29]. While this can enhance digital literacy in app-based research, the addition of blockchain features complicates these efforts [30-32]. User-centered design and user testing the comprehension of complex information are essential to uncover such challenges sooner.

Participants also revealed how differing privacy-by-design approaches can affect research participation. Even though prototype A had extensive explanatory content, participants’ perceptions were collectively better compared to prototype B, which lacked a formal informed consent process and emphasis on blockchain capabilities. Prototype A notably trended toward a higher level of perceived privacy and trust and a lower level of privacy concern and perceived privacy risk compared to prototype B. Although we were unable to detect statistically significant differences between means across all constructs of both prototypes, we were able to show the practical significance of these constructs (that most are highly correlative) on participants’ behavioral intention to use the app.

Second, we found that what was most valuable to women participants in app-based research was consistent with qualms already expressed in conventional research. Wilkins et al [33] found through a national survey that research participants preferentially valued receiving research results and updates on how their data were used, which is also associated with a higher likelihood of trusting researchers. Perceived usefulness, particularly the value of the research to women’s health and oneself was an important driver of participation in the proposed research apps. Participants also periodically made appraisals of that value (eg, progress, outcomes, impact) to determine their continued participation. This demonstrates that one’s decision to participate in research is a dynamic process that requires constant cultivation, especially if a study calls for long-term participation.

To meet these fundamental participant needs and expectations, design, and research teams need to prioritize efforts in building channels of communication with participants for the “return of value” and information [33]. Rather than acquiesce to the usual unidirectional data flow from participants to researchers, the return of study findings or data analytics to participants can make great strides in promoting their prolonged engagement and participation. There are also ethical benefits of providing data back to participants from the standpoint of respecting their autonomy and fairness of research benefits. If this option is not feasible, providing compensation for participants’ time and contributions is another way to motivate their participation. Otherwise, the study runs the risk of losing consistent contributions of quality data for women’s health research and wasting already expended efforts and resources.

Third, data privacy and security concerns were not at the forefront of women participants’ minds. Given the stigma in reproductive and sexual health, we had initial concerns about the sensitivity and extent of data elements requested from participants [9,34]. The list of data elements was originally drawn from Apple Health’s “Reproductive Health” category, which has since been rebranded to “Cycle Tracking” with a considerably longer list of data elements. Surprisingly, participants were open and willing, without an overly expressed sense of stigma, to share these data with researchers. While this may be partially attributed to the qualities of our recruited population, such as their interest and willingness to participate in research, it may also point to a larger cultural or generational shift toward women’s health issues. An important caveat is that these attitudes may have evolved considering the post-*Dobbs* era and threats to the privacy and safety of women [12,13].

This willingness to contribute data is predicated on an inherent trust in systems, particularly with affiliated academic institutions and the protections they have in place. Despite potentially having control over data sharing and establishing ownership over one’s data via blockchain capabilities, participants were generally satisfied with fewer technical safeguards, such as statements about privacy and security protections. Although participants may be content with deferring the safeguard of their data and interests to the study and its partnering entities, this does not absolve research stakeholders from further reinforcing their security protections. Participants’ trust in academic institutions is fragile and research stakeholders have a responsibility to minimize threats to its credibility. Trust, once lost, is very hard to rebuild. Research stakeholders need to remain vigilant in upholding strong data privacy and security practices regardless of participants’ changing attitudes toward data contribution.

Finally, even though our evaluation suggests a wavering level of perceived usefulness and comprehension with blockchain technology among women participants, it does not undercut the technology’s actual capabilities and value to society. While blockchain-enhanced features in the prototypes did not impact participant perceptions and attitudes toward use as much as we had expected, the technology encompasses societal values (eg, privacy, democratization, transparency, trustworthiness, reliability) that will endure in their relevance to digital health research [35]. Nonetheless, the technical benefits of blockchain will not be sufficient in affecting digital health research without detailed work on comprehension, explainability, and sustainable implementation.

### Limitations

The study had several limitations. First, we recruited women from the Xlab human subject pool, which consisted of students, staff, and alumni from UC Berkeley. This exposed our study to potential selection bias, where participants may not be representative of the general population. The sociodemographic characteristics of the UC Berkeley community, and the San Francisco Bay Area at large, are different (eg, higher household income and educational attainment) compared to the rest of the United States [36-38]. Also, those who volunteer to participate in digital health research have been found to have different characteristics compared to the general population [39]. To mitigate bias, we used purposive sampling to create a more representative sample across sociodemographic characteristics and levels of study topic literacy or familiarity.

Second, our study was partially limited by its small sample size of 16 participants, which reduced the statistical power of our perception survey analyses. Although we were unable to find a statistical effect between construct means of prototypes A and B, the sample size yielded insights into their practical significance. A sample size of 16 participants, however, is sufficient for usability testing, where approximately 5 users are considered adequate to reveal major heuristic violations. For the interviews, we reached a level of thematic saturation with the given sample size.

Finally, the prototypes were not technically functional, which limited participants’ assessment of their full capability. We also did not fully design and operate prototype features that were deemed not critical to the research study workflow. While we cannot determine the actual use of a live research study app, our use of mixed methods helped triangulate and identify positive features and processes that can enhance research participation.

### Conclusions

A privacy-by-design approach in digital health research proves to be promising for research participation. This study revealed that blockchain-enhanced capabilities in health research apps were ultimately secondary in value to participant fundamental expectations (eg, communication with researchers and return of findings) with health research. Hence, the use of novel technological solutions may not be sufficient to significantly affect participation. However, with growing data privacy and security concerns, it remains prudent for organizations to explore effective and sustainable integration of blockchain capabilities.

## Appendix

Multimedia Appendix 1 Design features of prototype A vs prototype B.

Multimedia Appendix 2 Screenshots of prototype A vs prototype B (selection).

Multimedia Appendix 3 Data collection instruments.

Multimedia Appendix 4 Participant user IDs and grouping details (N=16).

Multimedia Appendix 5 Overview of themes organized to technology acceptance model constructs and recommendations for prototype improvement.

## Abbreviations

AMI: area median income
CHERRIES: Checklist for Reporting Results of Internet E-Surveys
COREQ: Consolidated Criteria for Reporting Qualitative Studies
eIC: electronic informed consent
mHealth: mobile health
SES: socioeconomic status
SUS: System Usability Scale
TAM: technology acceptance model
UC Berkeley: University of California, Berkeley
Xlab: Experimental Social Science Laboratory
