# Supplementary material for: Participant Evaluation of Blockchain-Enhanced Women’s Health Research Apps: Mixed Methods Experimental Study
Source: JMIR Mhealth Uhealth. 2025 Mar 25;13:e65747. doi: 10.2196/65747 (PMC11979549; doi:10.2196/65747)
Supplement: Multimedia Appendix 1 [file mhealth_v13i1e65747_app1.pdf]

Multimedia Appendix 1. Design features of Prototype A vs Prototype B.

Summary Description of Features	Prototype A	Prototype B
**Account Creation and Registration**		
Creation of a "public key" for the user, which does not require any identifiable information and allows the user to participate in a pseudonymous manner with app stakeholders.	✓	✓
Requires setting up a 12-word phrase to enter the app		✓
Provide "Emergency Medical Record" section with some identifiable information (e.g., name, date of birth, gender)		Optional
**Enrollment into Study**		
Explanatory section on Bitmark Inc and the capabilities of a blockchain-enhanced health research infrastructure	✓	
Complete eIC[a] process at onboarding stage of research study workflow	✓	
**Health Data Contribution**		
Study activities and tasks	✓	✓
Integration with HealthKit for accessing data	✓	✓
All study data are "actively" contributed by the participant and transferred directly to the researcher only	✓	
Requires touch ID or pin-number authorization to initiate each "active" data transfer of study data	✓	
Visual ledger-like representation of metadata tags or "titling" of data onto the Bitmark blockchain	✓	
Authorized Health app study data are "passively" abstracted from the participant phone and transferred to a "data vault" for aggregate analyses by the larger research community		✓

[a]eIC: electronic informed consent
