# Supplementary material for: Participant Evaluation of Blockchain-Enhanced Women’s Health Research Apps: Mixed Methods Experimental Study
Source: JMIR Mhealth Uhealth. 2025 Mar 25;13:e65747. doi: 10.2196/65747 (PMC11979549; doi:10.2196/65747)
Supplement: Multimedia Appendix 2 [file mhealth_v13i1e65747_app2.pdf]

**Multimedia Appendix 2.** Screenshots of Prototype A vs Prototype B (selection).

Prototype A's landing page after account creation (A). Participants had difficulty finding the research study to join, which was nested under the "Donations" tab. Once they found the location of the study (B), they were able to join with minimal difficulty.

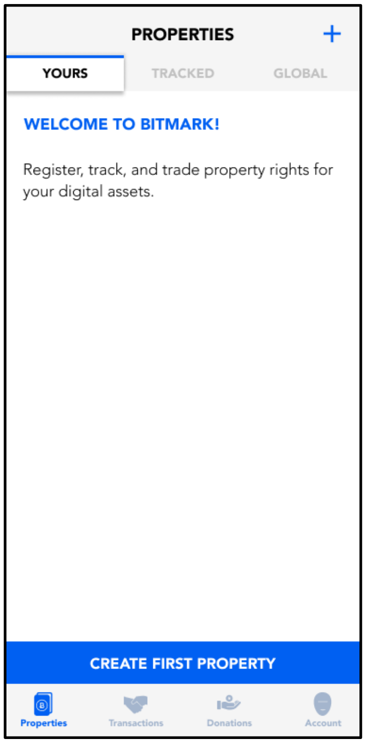


*A. Landing page after account creation in Prototype A.*

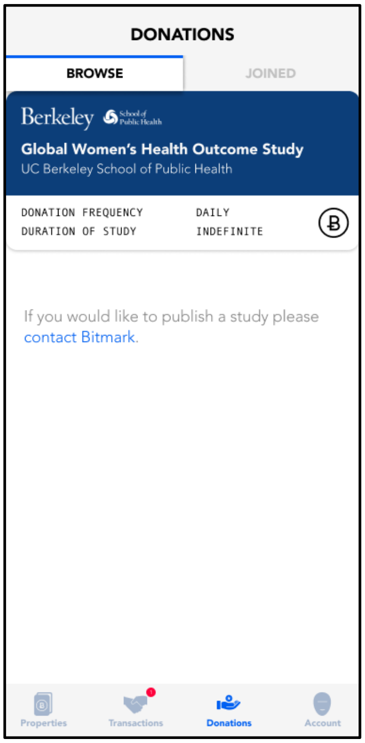


*B. Page with the research study is within the "Donations" tab.*

Prototype B's landing page after account creation (C). Participants had less difficulty finding the research study since it was located on the same screen.

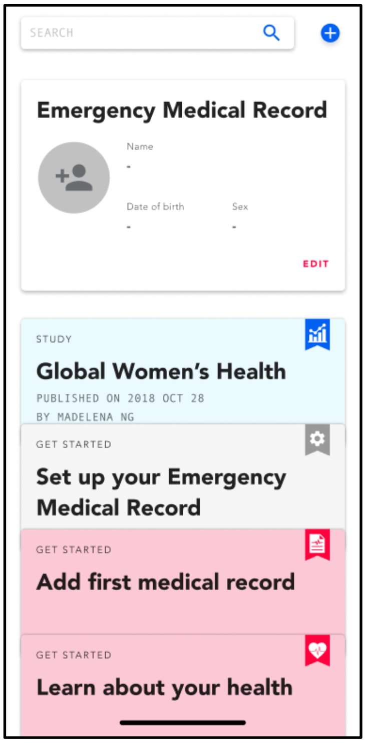


*C. Landing page after account creation in Prototype B. Note that the research study is on the same screen.*

For both Prototype A and B, participants were able to complete study tasks and activities (D, E), as well as authorize categories of women's health data to contribute to the research study (F).

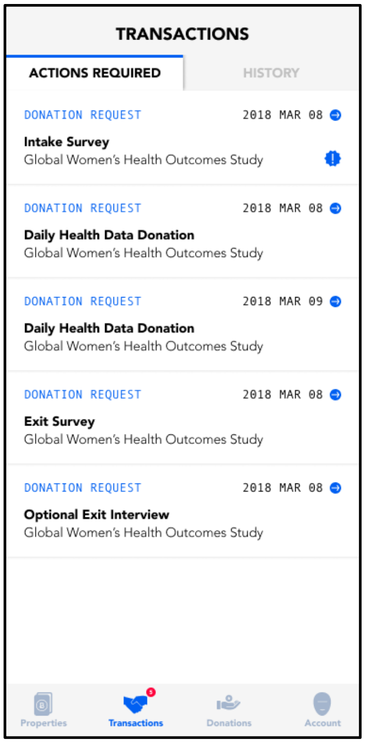


*D. Study activity and tasks in Prototype A.*

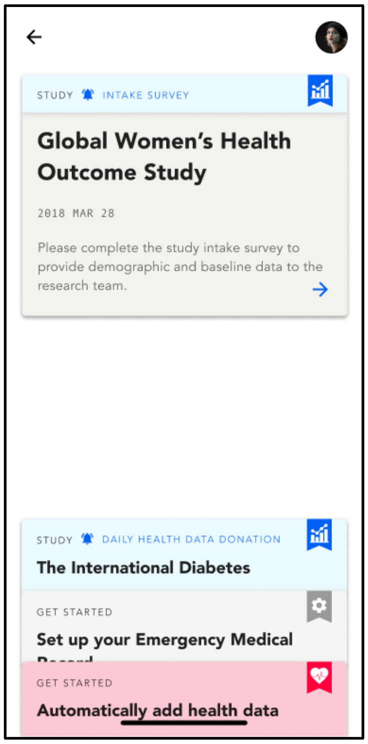


*E. Study activities and tasks in Prototype B.*

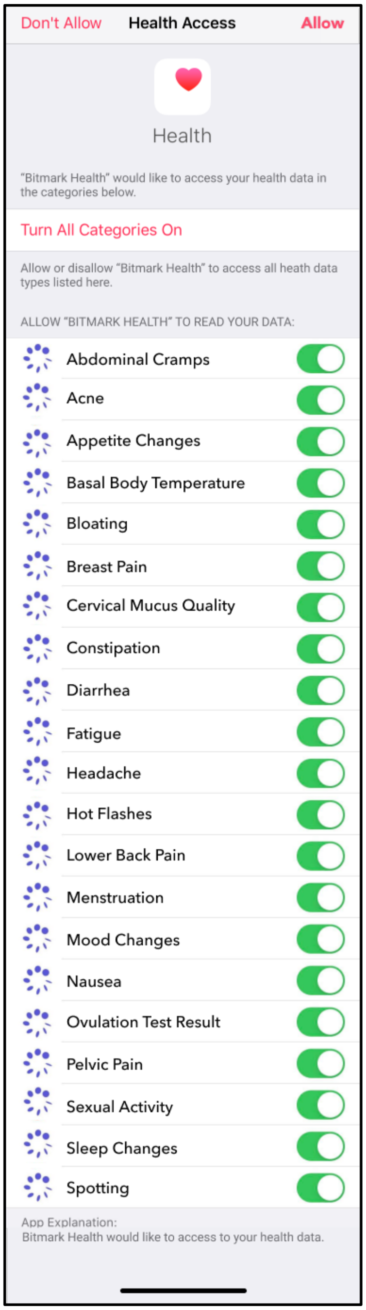


*F. Participant authorizes the contribution of health data from the Health app.*
