# Supplementary material for: Participant Evaluation of Blockchain-Enhanced Women’s Health Research Apps: Mixed Methods Experimental Study
Source: JMIR Mhealth Uhealth. 2025 Mar 25;13:e65747. doi: 10.2196/65747 (PMC11979549; doi:10.2196/65747)
Supplement: Multimedia Appendix 3 [file mhealth_v13i1e65747_app3.pdf]

Multimedia Appendix 3. Data collection instruments.

I. <u>Background Questionnaire</u>

1. Age range:
    a. 18-29
    b. 30-39
    c. 40-49
    d. 50-59
    e. 60+
2. Race (check all that apply):
    a. White
    b. Black or African American
    c. Asian
    d. American Indian or Alaska Native
    e. Native Hawaiian or Pacific Islander
    f. Other
3. Ethnicity:
    a. Hispanic or Latino
    b. Not Hispanic or Latino
4. Highest education level:
    a. Doctoral or professional degree
    b. Master's degree
    c. Bachelor's degree
    d. Associate's degree
    e. Some college, no degree
    f. High school diploma or equivalent
    g. No formal educational credential
5. Yearly household income: __________
6. Number of people in household (including yourself): _________
7. How long have you been using a smartphone?
    a. Less than a year
    b. 1-2 years
    c. 3-5 years
    d. More than 5 years
8. On average, how many hours a day do you use your smartphone?
    a. Less than an hour
    b. 1-2 hours
    c. 3-4 hours
    d. 5+ hours
9. Have you ever participated in a mobile or app-based health research study?
    a. Yes → Please list the study name(s).
    b. No
10. Are you a user of any women’s health-related apps (e.g., period tracker, fertility)?
    a. Yes → Please list the app name(s).
    b. No

II. <u>Think-aloud Protocol</u>

Think-aloud protocols involve participants thinking aloud as they are performing a set of specified tasks. Participants are asked to say whatever comes into their mind as they complete the task. This might include what they are looking at, thinking, doing, and feeling.

We will use this protocol to assess the usability of the mobile digital study and user thought processes when completing tasks.

<u>Assessing Key Steps of Digital Research Participation:</u>
List of Tasks—

1. Create an account/register into the app
2. Join a women's health research study.
3. Contribute health data to the study.

III. <u>Perceptions Survey</u>

Participants are asked to score the following items with responses that range from Strongly Disagree to Strongly Agree:

Type/Construct	Items	Corresponding Survey Description	Source
Privacy concern *(Scale of 1-5)*	PCON1	I am concerned that the information I submit to this research study app could be misused.	Adapted from Xu, 2011 [19]
	PCON2	I am concerned that others can find private and personal information about me from this research study app.	
	PCON3	I am concerned about providing personal information to this research study app because of what others might do with it.	
	PCON4	I am concerned about providing personal information to this research study app because it could be used in a way I did not foresee.	
Perceived privacy risk *(Scale of 1-7)*	RISK1	In general, it would be risky to give personal information to this research study app.	Adapted from Dinev, 2013 [20] and Malhotra, 2004 [21]
	RISK2	There would be high potential for privacy loss associated with giving personal information to this research study app.	
	RISK3	Personal information could be inappropriately used by this research study app.	
	RISK4	Providing this research study app with my personal information would involve many unexpected problems.	
Perceived control *(Scale of 1-7)*	PCTL1	I believe I have control over who can get access to my personal information collected by this research study app.	Adapted from Xu, 2011 [19]
	PCTL2	I think I have control over what personal information is released by this research study app.	
	PCTL3	I believe I have control over how personal information is used by this research study app.	
	PCTL4	I believe I can control my personal information provided to this research study app.	
Perceived ownership *(Scale of 1-7)*	*The following questions deal with the 'sense of ownership' that you feel for your personal health data.*		Adapted from Van Dyne and Pierce, 2004 [22]
	OWN1	This is MY data.	
	OWN2	I sense that this is MY data.	
	OWN3	I feel a high degree of personal ownership for this data.	
	OWN4	I feel like I own my data.	
	OWN5	Most of the people I know feel as though they own their data.	
	OWN6	It is hard for me to think about this data as MINE.	
Perceived privacy *(Scale of 1-7)*	*When you answer the following questions about your privacy, please think about the limited access that clinical researchers have to your personal information.*		Adapted from Dinev, 2013 [20]
	PRIV1	I feel I have enough privacy when I use this research study app.	
	PRIV2	I am comfortable with the amount of privacy I have when using this research study app.	
	PRIV3	I think my online privacy is preserved when I use this research study app.	

Trust<br>*(Scale of 1-7)*	TRU1	The research study app’s policy with respect to how they will share my personal information with third parties makes me feel the study is trustworthy.	Adapted from Liu, 2004 [18]
	TRU2	The research study policy on how it would use any personal information about me makes me feel that the study is trustworthy.	
	TRU3	The ability to access my personal information to ensure that it is accurate and complete makes me feel that the study is trustworthy.	
	TRU4	The research study app’s security policy makes me feel that the study is trustworthy.	
	TRU5	The research study app’s informed consent makes me feel that the study is trustworthy.	
	TRU6	The research study app’s design features makes me feel that the study is trustworthy.	
	TRU7	The research study app’s level of encryption and other security measures makes me feel that the study is trustworthy.	
	TRU8	The research study app’s policy concerning the safeguard of personal information collection makes me feel this study is trustworthy.	
Usability<br>*(Scale of 1-5)*	*To assess overall usability and user satisfaction:*		Adapted from Brooke, 1986 [23]
	SUS1	I think that I would like to use this research study app frequently.	
	SUS2	I found the research study app unnecessarily complex.	
	SUS3	I thought the research study app was easy to use.	
	SUS4	I think that I would need the support of a technical person to be able to use this research study app.	
	SUS5	I found the various functions in this research study app were well integrated.	
	SUS6	I thought there was too much inconsistency in this research study app.	
	SUS7	I would imagine that most people would learn to use this research study app very quickly.	
	SUS8	I found the research study app very cumbersome to use.	
	SUS9	I felt very confident using the research study app.	
	SUS10	I needed to learn a lot of things before I could get going with this research study app.	
Behavior intention to use the system<br>*(Scale of 1-5)*	*To what extent do you intend to do the following activities?*		N/A
	INT1	I intend to download the research study app.	
	INT2	I intend to give informed consent.	
	INT3	I intend to participate in research activities such as completing surveys.	
	INT4	I intend to participate in research activities such as donating my digital health data.	

IV. <u>Semi-structured Interview Guide</u>

To gauge what design features worked/did not work, thoughts on contributing women's health data, thoughts on blockchain technology, thoughts on design feature improvement. Additional questions and probes may arise during the interviews.

1. How do you feel about contributing the following women's health data to a digital research study/Prototype A/Prototype B?
   - Abdominal Cramps
   - Acne
   - Appetite Changes
   - Basal Body Temperature
   - Bloating
   - Breast Pain
   - Cervical Mucus Quality
   - Constipation
   - Diarrhea
   - Fatigue
   - Headache
   - Hot Flashes
   - Lower Back Pain
   - Menstruation
   - Mood Changes
   - Nausea
   - Ovulation Test Result
   - Pelvic Pain
   - Sexual Activity
   - Sleep Changes
   - Spotting
2. Do you foresee yourself participating in either Prototype A or Prototype B consistently? Why or why not?
3. Were there any design features that were particularly engaging or of interest? What function do you think they served?
4. What is your understanding of, or familiarity with, blockchain technology?
5. Were you aware of any design features in Prototype A or Prototype B that indicated the incorporation of such technology?
6. Would the incorporation of this technology make a difference in your long-term research participation/data donation?
7. What design features or other additional components would make a difference in your long-term research participation/data donation?
