# Supplementary material for: Participant Evaluation of Blockchain-Enhanced Women’s Health Research Apps: Mixed Methods Experimental Study
Source: JMIR Mhealth Uhealth. 2025 Mar 25;13:e65747. doi: 10.2196/65747 (PMC11979549; doi:10.2196/65747)
Supplement: Multimedia Appendix 4 [file mhealth_v13i1e65747_app4.pdf]

Multimedia Appendix 4. Participant user IDs and grouping details (N=16).

Groups[a]	Ever joined a digital study or used a women's health app?	
	Yes	No
White, non-Hispanic women with higher SES[b]	[01] A, 18-29, 110%, Bachelor's<br>[02] B, 30-39, 140%, Master's	[03] A, 18-29, >200%, Bachelor's<br>[04] B, 30-39, >200%, Doctoral
White, non-Hispanic women with lower SES	[05] A, 18-29, 85%, some college (no degree)<br>[06] B, 18-29, 70%, some college (no degree)	[07] A, 40-49, 20%, Bachelor's<br>[08] B, 30-39, 70%, Bachelor's
Women of color with higher SES	[09] A, 30-39, 200%, Doctoral<br>[10] B, 40-49, 80%, Bachelor's	[11] A, 30-39, 150%, Master's<br>[12] B, 18-29, >200%, Bachelor's
Women of color with lower SES	[13] A, 18-29, 70%, Associate's<br>[14] B, 18-29, 30%, some college (no degree)	[15] A, 18-29, 40%, some college (no degree)<br>[16] B, 30-39, 20%, Associate's

[a]Each line within the table cells corresponds to: [Participant ID] randomized to test prototype A or B first, age group, % Area Median Income, highest education level
[b]SES: socioeconomic status
