# Supplementary material for: Participant Evaluation of Blockchain-Enhanced Women’s Health Research Apps: Mixed Methods Experimental Study
Source: JMIR Mhealth Uhealth. 2025 Mar 25;13:e65747. doi: 10.2196/65747 (PMC11979549; doi:10.2196/65747)
Supplement: Multimedia Appendix 5 [file mhealth_v13i1e65747_app5.pdf]

Multimedia Appendix 5. Overview of themes organized to TAM constructs and recommendations for prototype improvement.

Construct and theme	Facilitator	Barrier	Recommendations
**Perceived ease of use**			
Intuitive navigation	Ease of health data contribution	Unclear account creation and location of research study within the app	• Provide additional clarification on what an "account" entails in a pseudonymous digital health research environment.<br>• Nest the research study in a logical and visible area of the app (eg, designated "Study" or "Research" tab or section).
Comprehension	Formal eIC process	Too much complex information and terms	• Have an apparent study eIC but present the most critical information with consideration of inclusivity (e.g., digital literacy, readability).
**Perceived usefulness**			
Value of research study to women's health field	Belief the study can advance women's health research	None noted	• Incorporate more encouraging content and reminders about how the health data contributed can advance the field of women's health and address gender inequities in biomedical research.
Value of research study to self	Extent of direct or indirect benefits	None noted	• Consider issuing financial compensation for participation or return relevant findings to participants.
	Updates about research study progress and how data contributed help to advance study goals	None noted	• Add a page or tab in the app where researchers can generate and deliver regular updates about study progress and discoveries to participants.
Value of blockchain features for participation	Belief that blockchain is trustworthy and can enhance privacy and security	Limited understanding of blockchain hinders full assessment of its value and relevance	• Provide additional educational content. As blockchain becomes more widely adopted, understanding its capabilities and relevance may impact research participation.
